# Rumen-protected glucose supplementation alters fecal microbiota and its metabolic profiles in early lactation dairy cows

**DOI:** 10.3389/fmicb.2022.1034675

**Published:** 2022-12-02

**Authors:** Yapin Wang, Yiguang Zhao, Xuemei Nan, Yue Wang, Meng Cai, Linshu Jiang, Qingyao Luo, Benhai Xiong

**Affiliations:** ^1^State Key Laboratory of Animal Nutrition, Institute of Animal Science, Chinese Academy of Agricultural Sciences, Beijing, China; ^2^Beijing Key Laboratory for Dairy Cow Nutrition, Beijing University of Agriculture, Beijing, China

**Keywords:** negative energy balance, rumen-protected glucose, fecal microbiota, metabolite, dairy cows

## Abstract

**Introduction:**

Negative energy balance (NEB) is the pathological basis of metabolic disorders in early lactation dairy cows. Rumen-protected glucose (RPG) is a feed additive to relieve NEB of cows in early lactation. The aims of the current study were to evaluate the impact of different doses of RPG supply on fecal microbiota and metabolome in early lactation dairy cows, and their correlation with each other.

**Methods:**

A total of 24 multiparous Holstein dairy cows in early lactation were randomly assigned to one of four treatments for the first 35 days of the early lactation period, as follows: control group, a basal diet without RPG (CON); low RPG, a basal diet plus 200 g/d RPG (LRPG); medium RPG, a basal diet plus 350 g/d RPG (MRPG); or HRPG, high RPG, a basal diet plus 500 g/d RPG (HRPG). After 35 days, fecal samples were obtained from cows in all groups individually and using 16S rRNA gene sequencing to evaluate their microbiotas, while their metabolites were evaluated through metabolomics.

**Results:**

As expected, *Firmicutes* and *Bacteroidetes* were the core bacteria phyla. After RPG supplementation, there were an increase in *Firmicutes* and a decrease in *Bacteroidetes*. MRPG increased the relative abundance of cellulolytic bacteria, including *Ruminococcaceae_UCG-005*, *Lachnospiraceae_UCG-008*, *Lachnospiraceae_FCS020_group*, and *Ruminiclostridium_9*, while it decreased the relative abundance of *Alistipes*, *Prevotellaceae_UCG-003*, and *Dorea*. RPG supplementation could regulate the carbohydrate metabolism and amino acid metabolism pathway significantly and relieve lipolysis in dairy cows. Correlation analysis of fecal microbiome and metabolome showed that some major differential bacteria were the crucial contributors to differential metabolites.

**Conclusion:**

In conclusion, RPG supplementation can affect the fecal microbial components and microbial metabolism, and 350 g RPG might be the ideal dose as a daily supplement.

## Introduction

Accompanied by the decrease in dry matter intake (DMI) around parturition and the increased energy requirements for milk in early lactation, cows are prone to negative energy balance (NEB), leading to a series of metabolic disorders such as ketosis and fatty liver (Pérez-Báez et al., 2019a,b). The essence of NEB is the lack of glucose, while the bacterial transformation of dietary components may play key roles in host health and disease (Zhang and Davies, 2016), so the potential of manipulating the microbiome and meeting livestock challenges through dietary interventions have recently emerged as promising new technologies (Huws et al., 2018). Extensive degradation of directly supplemented glucose in the rumen via microbial degradation substantially lowers the post-ruminal availability of dietary glucose. Rumen-protected glucose (RPG) can escape rumen fermentation partly and play a role in the intestine under the coating of hydrogenated fat.

The gut microbiota produces a large number of bioactive metabolites through fermented feed, which plays a crucial role in regulating key physiological processes, like host metabolism and immune response (Thursby and Juge, 2017). These coordinated processes facilitate the growth and development of dairy cows (Malmuthuge and Guan, 2017). Recently, some research revealed that diet changes greatly influence the intestinal microbiota of cattle (Shanks et al., 2011; Faulkner et al., 2017; Lourenco et al., 2020), and the fecal microbiome may serve as a means for assessing intestinal (Faulkner et al., 2017). The fecal microbiome of cows plays an important part not only in animal development and performance but also in food safety, pathogen shedding, and the performance of fecal contamination detection methods (Shanks et al., 2011). Diet is considered as one of the main drivers that shape the gut microbiota throughout life time. Intestinal bacteria play an important part in maintaining immune and metabolic homeostasis and protecting against pathogens. Altered gut bacterial composition and even dysregulation are implicated in the pathogenesis of many inflammatory diseases and infections (Thursby and Juge, 2017). A previous study uncovered that dietary RPG supplementation not only promoted epithelial metabolism but also improved immune homeostasis in the ileum (Zhang et al., 2019). However, as a feed additive that plays a role in the hindgut, whether and how RPG supplementation has an effect on the flora and metabolism of the hindgut microorganisms remains unknown.

Metabolomics constitutes a powerful avenue for the functional characterization of the gut microbiota and its interactions with the host (Marcobal et al., 2013). The application of metabolomics approaches has greatly advanced our understanding of the mechanisms that link the composition and activity of the gut microbiote to health and disease phenotypes (Thursby and Juge, 2017). Our previous work used metabolomics technology to reveal that RPG had a positive effect on dairy cattle serum metabolism and conventional serum biochemical indicators of energy balance. As far as we know, no studies were performed to investigate the effects of different doses of RPG supplementation on fecal microbiota and metabolites in early lactation dairy cows. Therefore, it was our objective to evaluate the dynamic profile changes and interactions of fecal microbiota and metabolites in early lactation dairy cows by supplementing different doses of RPG. We hypothesized that RPG supplementation can improve the structure of intestinal microbiota, alleviate lipid metabolism of intestinal microbiota, and provide more glucose for intestinal absorption and utilization. To address this hypothesis, we employed 16S rRNA gene sequencing along with metabolomics approach to exhaustively characterize the structural and metabolic changes in the gut microbiota elicited by experimental treatments.

## Materials and methods

Protocols for this experiment were reviewed and approved by the Animal Ethics Committee of the Chinese Academy of Agricultural Sciences (Beijing, China) (Approval Number: IAS2019-54) and were under the academy’s guidelines for animal research.

### Animals, diets, and experimental design

The dairy farm is located in Beijing, China, and is affiliated with Beijing Sunlon Livestock Development Ltd. All diets were formulated to meet National Research Council [NRC] (2001) dairy predicted requirements. Nutrient composition of the basal diet during the experiment is shown in Supplementary Table 1. During the experiment, cows were milked three times daily at 06:00, 13:00, and 20:00 and fed three times daily at 07:00, 14:00, and 21:00, respectively. Cows were housed in individual stalls, where water was available at all times.

Multiparous Holstein cows in early lactation (*n* = 24) were grouped according to their parity (2.92 ± 1.19) and milk yield (35.78 ± 7.90 kg/d) and assigned randomly to receive control diet (CON, *n* = 6), low RPG diet (LRPG, *n* = 6), medium RPG diet (MRPG, *n* = 6), or high RPG diet (HRPG, *n* = 6) group in a randomized block design. Supplemental RPG was fed twice daily as a top dress on the basal diet from the calving date to 35 days postpartum with the first 7 days for adaptation. The dosage of RPG was based on the manufacturer’s recommendation (200–500 g/cow/day) and a previous study which reported 200 g of supplemented RPG was insufficient to meet the elevated energy requirements associated with increased milk production which reported 200 g of supplemented RPG was insufficient to meet the elevated energy requirements associated with increased milk production (Li et al., 2019; Wang et al., 2020). Therefore, cows were supplemented with 0, 200, 350, or 500 g RPG per cow per day, respectively. The commercially sourced RPG is in the form of small beads made by mass with 45% glucose as the core, 45% hydrogenated fat as the coating, and 10% water. As such, 0, 90, 157.5, or 225 g of glucose supplement per day was received by cows from CON, LRPG, MRPG, or HRPG group, respectively.

### Fecal sampling and storage

At the end of the experiment (day 35), fecal samples were collected by digital rectal palpation before midday feeding according to institutional animal care guidelines. Immediately after collection, a subset of the fecal samples were immersed in liquid nitrogen for freezing and then stored at −80°C for further DNA extraction, and part of the samples used for metabolomics analysis were first filtered through four layers of cheesecloth, followed by the same procedures. Unfortunately, a fecal sample used to determine the 16S rRNA was damaged.

### DNA extraction and sequencing

Using the E.Z.N.A.^®^ soil DNA Kit (Omega Bio-tek, Norcross, GA, USA) to extract microbial genomic DNA from fecal samples according to manufacturer’s instructions. The DNA extract was checked on 1% agarose gel, and DNA concentration and purity were determined with NanoDrop 2000 UV-vis spectrophotometer (Thermo Fisher Scientific, Wilmington, USA). After extracting the total DNA of the sample, primers were designed according to the conserved region, the hypervariable region V3-V4 of the bacterial 16S rRNA gene was amplified with primer pairs 338F (5′-ACTCCTACGGGAGGCAGCAG-3′) and 806R (5′-GGACTACHVGGGTWTCTAAT-3′) by an ABI Gene Amp^®^ 9700 PCR thermocycler (ABI, CA, USA) (Zhang et al., 2017), and sequencing adaptors were added to the end of primers. The amplicons were purified, quantified, and homogenized to obtain a sequencing library; then library QC was performed and the qualified library was sequenced on Illumina HiSeq 2500. The original image data files got by high-throughput sequencing of sequencing platforms, such as Illumina MiSeq were converted into Sequenced Reads by Base Calling analysis, and the results were stored in FASTQ referred to as fq format file, which contains sequence information of reads and their corresponding sequencing quality information.

### Bioinformatics workflow

Data preprocessing: According to the overlap relation between PE reads, merge the paired-ends sequence data obtained from Miseq sequencing into tags of one sequence, then perform quality control and filtering for reads quality and Merge effect. There are three main steps as follows: (1) PE reads merge: FLASH (Version 1.2.7) software is used to merge reads through overlap, the obtained merged sequences are Raw Tags; (2) Tags filtering: Use Trimmomatic (Version 0.33) software to filter merged Raw Tags to get high quality processed reads; (3) Remove Chimera: Use UCHIME (Version 4.2) software to identify and remove chimera sequences to get Effective Tags. Bioinformatics content: Divide operational taxonomic units (OTU), diversity, and difference analysis. All the raw reads were submitted to the NCBI Sequence Read Archive (SRA)^1^ database (Accession Number: PRJNA657329).

OTU is Operational Taxonomic Unit. Perform OTU classification for all sequences according to different similarity levels. Generally speaking, when the similarity between sequences is higher than 97%, it can be defined as an OTU, and each OTU corresponds to a representative sequence. Use Usearch software (Edgar, 2013) then perform taxonomic annotation for OTU on the basis of SILVA (Bacteria) and UNITEUNITE (fungi) taxonomic database. The count of OTU of each sample was obtained at 97% similarity level. Combined with the species represented by OTU, shared microorganisms in different environments can be found. In order to obtain the species classification information of each OTU, the representative sequences of the OTU can be aligned to the microbial reference database, and then the community composition of each sample at each level (phylum, class, order, family, genus, and species) was counted.

Use QIIME software to generate the composition of the microbial community, at varying taxonomic levels, which was graphed in R (detail the package used and the version of R used). Alpha diversity reflects the species richness and the species diversity of the individual sample. Use Mothur (Version 1.30) software to investigate the Alpha diversity index of samples. The Shannon diversity rarefaction curve is made by Mothur software and R, Shannon indexes of sequencing amount under different sequencing depths are also used. Use QIIME software to perform Beta diversity analysis to compare the similarity of species diversity between different samples. Non-metric multi-dimensional scaling (NMDS) can simplify the research objects from multi-dimensional space to low-dimensional space for positioning, analysis, and classification. Significant difference analysis between groups is mainly used to find biomarkers with statistical differences between different groups. According to the established Biomarker screening criteria [line discriminant analysis (LDA) score > 2], the eligible biomarkers are identified and presented as icons. Line discriminant analysis effect size (LEfSe) can find Biomarkers with statistical difference between different groups (Segata et al., 2011), by combining the statistical significance of the Kruskal-Wallis tests with additional tests to assess biological consistency and effect relevance.

### Metabolomics processing

Fecal samples were layered for 1 h at room temperature and then centrifuged at 3,000 g for 10 min at 4^°^C. Before analysis, the supernatant was added with 400 μL of ice-cold methanol/water (4:1, v/v) and then ground with high-throughput tissue grinder at a low temperature. After vertexing, samples were sonicated three times for 10 min on ice, followed by treated at −20^°^C for 30 min. Subsequently, after a centrifugation at 13,000 *g*, 4^°^C for 15 min, all the samples supernatant were collected and transferred to injection vials for further liquid chromatography-mass spectrometry analysis (LC-MS).

Fecal metabolites were analyzed using Ultra-performance liquid chromatography (UPLC) coupled with a Triple time-of-flight (TOF) system (AB SCIEX, Framingham, USA). The chromatographic separation was performed on a BEH C18 column at 40^°^C (100 mm × 2.1 mm, 1.7 μm, Waters, Milford, USA). The mobile phase consisted of 0.1% formic acid aqueous solution (solvent A) and acetonitrile/isopropanol (v/v, 1:1) solution with 0.1% formic acid (solvent B). The injection volume was 20 μL and the elution gradient was shown in Supplementary Table 2.

Mass spectrometry was performed on a Triple TOF mass spectrometer, and the electrospray ionization source operated simultaneously in positive and negative-ion modes. The capillary voltage was 1.0 kV, the injection voltage was 40 V, and the collision energy was 6 eV. Source temperature was 120^°^C, and desolvation temperature was 500^°^C. Gas flow rate was 900 L/h. Mass data were collected between 50 and 1,000 m/z and the instrument resolution was 30,000.

### Metabolomics data analysis

Metabolomics data files were processed to multivariate statistical analysis using SIMCA-P 14.0 software (Umetrics, Umeå, Sweden). The preprocessing results were imported into the software package for log-transformation and pareto-scaling prior to performing multivariate analysis. The score plot of the unsupervised multivariate statistical principal component analysis (PCA) model was used to display the overall metabolic profile of all samples, which was used to reveal the similarities and dissimilarities among the inter-groups. Then partial least squares discriminant analysis (PLS-DA) was used to identify the overall differences in metabolic profiles between groups and to find metabolites with different abundance. PLS-DA is a widely used supervised multivariate classification model. Samples were specified and grouped during analysis, and the model will automatically add another data set Y. This method of model calculation forcibly classifies each component to facilitate the discovery of similarities and differences between different groups. For samples with insufficient differences between groups, the PLS-DA model may be more effective. In PLS-DA analysis, the value of variable importance in projection (VIP) score was greater than 1, which was the typical rule for selecting differential variables. The permutation test was a random sort method to evaluate the accuracy of the PLS-DA model. In order to prevent the model from over-fitting, 200 permutation tests were used to examine the fitting effect of the model. T-test combined with PLS-DA multivariate analysis was used to screen the metabolites with differential abundance between groups. The screening criteria were: VIP > 2 and *P* < 0.05.

### Correlations between microbial communities and fecal metabolites

By Spearman’s correlation analysis in R (version 3.2.4), correlation analysis of different metabolites with VIP > 2, *P* < 0.05, and differentially abundant microbial genera (*P* < 0.05 and relative abundance > 0.05% in at least one of the samples) were assessed. Spearman’s rank correlations were calculated using the Psych packages (FDR correct was embedded in the package) in R. Only connections with *p*-value below 0.05 and | r| > 0.4 were considered as statistically significant. These correlations were visualized using the Heatmap package in R and Cytoscape (version 2.8.2).

## Results

### Changes of fecal bacteria

Following Illumina sequencing, a total of the 1,807,144 raw reads were obtained in the four group samples. After paired-end reads were spliced and filtered, 1,760,224 processed reads were generated, accounting for 97% of the raw reads.

At least 44,129 processed reads were obtained from each sample, with an average of 76,531 processed reads. Rarefaction curves indicated that a high level of microbial diversity was obtained for subsequent analysis of treatments (Supplementary Figure 1). When the curve tends to be flat, it means the sequencing data are sufficient and the OTU species won’t grow with sequencing data growing (Supplementary Figure 1). In addition, OTU Coverage is counted, the higher its value is, the higher the probability of species measured in the sample is. The index reflects whether the sequencing result represents the actual situation of the microorganism in the sample. Similarly, the Good’s coverage value of all samples exceeded 99.7%, demonstrating the accuracy and reproducibility of the sequencing in this study.

**FIGURE 1:**
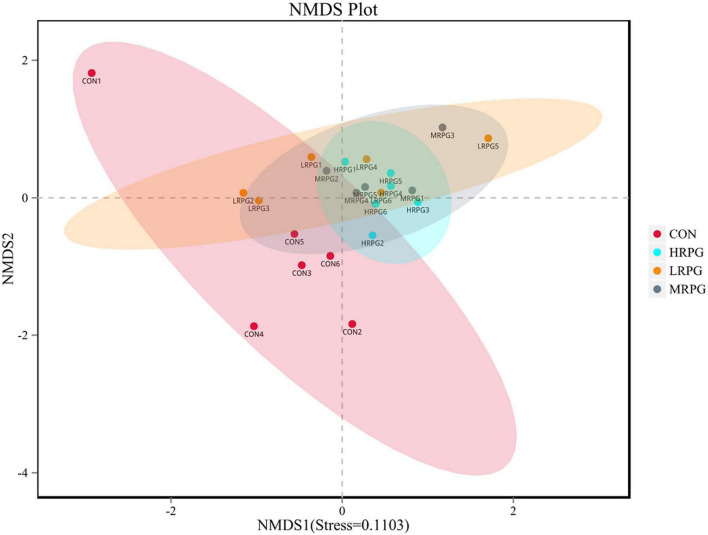
Non-metric multi-dimensional scaling (NMDS) analysis graph. Dots represent all samples; different colors represent different groups, and the distance between dots presents the difference; when stress is less than 0.2, it indicates that NMDS analysis has certain reliability. The closer the samples are on the coordinate diagram, the higher the similarity is.

The curve explains both the species richness and species evenness in the sample (Supplementary Figure 2). Rank abundance curves for all treatments were usually overlapping, which revealed that overall microbial diversity was similar. NMDS analysis result was shown as follows: the closer the samples are on the coordinate graph, the higher the similarity is. The treatment groups were separated from the control group (Figure 1). Chao1 and Ace indexes measure species richness, while larger Shannon index and smaller Simpson index indicate that the species diversity of the sample is higher (Grice et al., 2009). Alpha diversity analysis showed that there were no significant differences in diversity and richness between groups (Table 1).

**TABLE 1:** Alpha diversity index statistics of fecal microbiota.

Item	Treatments^a^				SEM^b^	*P*
	CON	LRPG	MRPG	HRPG		
OTU	689.67	678.83	681.60	693.50	28.50	0.793
ACE	703.62	697.07	691.68	707.51	20.22	0.552
Chao1	706.37	701.61	694.35	711.28	22.18	0.601
Simpson	0.03	0.05	0.058	0.06	0.02	0.140
Shannon	4.80	4.63	4.71	4.64	0.29	0.715

^a^ Treatments: CON, control group, a basal diet; LRPG, low RPG, a basal diet plus 200 g/d RPG; MRPG, medium RPG, a basal diet plus 350 g/d RPG; HRPG, high RPG, a basal diet plus 500 g/d RPG. ^b^ SEM, standard error of the mean.

**FIGURE 2:**
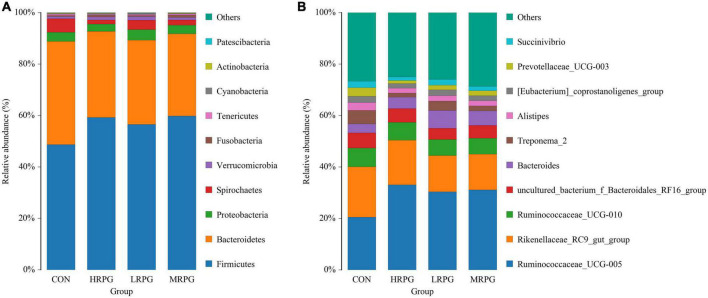
Histogram of species distribution of fecal microbiota. One color represents one specie, and the length of the color block presents the relative richness proportion of the species. For the best view, only species with the top 10 richness level will be shown, the rest species are combined as others in the chart, unclassified represents the species that has not been taxonomically annotated. The detailed species information can be found in the species richness table of the corresponding classification. **(A)** Phylum. **(B)** Genus.

In total, 12 bacterial phyla were identified in the fecal samples. Independent of the dose of RPG, *Firmicutes* and *Bacteroidetes* were the two predominant phyla in the dairy cow’s fecal samples. The combined abundance of these two phyla accounts for 90.46% of the entire microbial community. As shown in Figure 2A, the mean abundance levels of *Firmicutes* and *Bacteroidetes* are 55.9 ± 4.4% (mean ± standard error of the mean) and 34.6 ± 3.2%, respectively. Between them, the relative abundance of *Firmicutes* in CON was significantly lower than that in the three RPG supplementation groups (*P* = 0.001), while the *Bacteroidetes* were the opposite (*P* = 0.0004). The remaining bacterial phyla (e.g., *Proteobacteria*, *Spirochetes*, *Verrucomicrobia*, etc.) accounted for < 4% of the total sequences (Supplementary Table 3).

152 bacterial taxa were identified at the genus level through the analysis of microbiota compositions. Among these genus, *Ruminococcaceae_UCG-005* (28.6 ± 4.7%), *Rikenellaceae_RC9_gut_group* (16.2 ± 2.3%), *Ruminococcaceae _UCG-010* (6.75 ± 0.4%), *uncultured_bacterium_f_Bacteroidales _RF16_group* (5.2 ± 0.5%), *Bacteroides* (5.0 ± 1.27%), *Treponema_2* (3.2 ± 1.5%), and *Alistipes* (2.3 ± 0.4%) were considered as relatively high abundance genus (Figure 2B). Table 2 compared the fecal bacteria composition among the four groups. Among them, seven different genera bacteria were identified. *Ruminococcaceae_UCG-005* (*P* = 0.007), *Lachnospiraceae_UCG-008* (*P* = 0.005), *Lachnospiraceae_FCS020_group* (*P* = 0.020), and *Rumini- clostridium_9* (*P* = 0.012) were greater in MRPG and HRPG compared to CON. However, the relative abundance of *Alistipes* (*P* = 0.045), *Prevotellaceae_UCG-003* (*P* = 0.003), and *Dorea* (*P* = 0.023) was higher in CON than RPG supplementary groups (Table 2).

**TABLE 2:** Main microbiota (accounting for ≥ 0.05% of the total sequences in at least one of the samples) in the four groups (abundance of the genera is expressed as a percentage).

Phylum	Genus	Treatments^1^				SEM^2^	*P*
		CON	LRPG	MRPG	HRPG		
*Firmicutes*	*Ruminococcaceae_UCG-005*	20.60^b^	29.77^ab^	31.05^a^	32.76^a^	5.74	0.007
*Bacteroidetes*	*Rikenellaceae_RC9_gut_group*	19.61	14.11	13.95	17.15	3.51	0.033
*Bacteroidetes*	*Bacteroides*	3.40	6.76	5.60	4.37	2.54	0.147
*Spirochetes*	*Treponema_2*	5.41	3.72	2.01	1.63	3.87	0.333
*Bacteroidetes*	*Alistipes*	3.01^a^	2.13^ab^	2.03^ab^	1.88^b^	0.70	0.045
*Bacteroidetes*	*Prevotellaceae_UCG-003*	3.32^a^	1.78^b^	1.88^b^	1.14^b^	0.87	0.003
*Firmicutes*	*Lachnospiraceae_UCG-008*	0.24^b^	0.32^ab^	0.41^a^	0.34^ab^	0.07	0.005
*Firmicutes*	*Dorea*	0.20^a^	0.10^ab^	0.12^ab^	0.08^b^	0.07	0.023
*Firmicutes*	*Lachnospiraceae_FCS020_group*	0.08^b^	0.08^b^	0.11^a^	0.09^ab^	0.02	0.020
*Firmicutes*	*Ruminiclostridium_9*	0.06^ab^	0.04^b^	0.09^a^	0.07^ab^	0.02	0.012

^a,b,c^ Means with a row with different superscripts differ. ^1^Treatments: CON, control group, a basal diet; LRPG, low RPG, a basal diet plus 200 g/d RPG; MRPG, medium RPG, a basal diet plus 350 g/d RPG; HRPG, high RPG, a basal diet plus 500 g/d RPG. ^2^ SEM, standard error of the mean.

**FIGURE 3:**
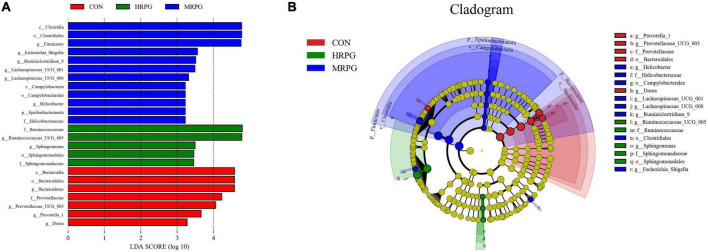
Line discriminant analysis effect size (LEfSe) analysis of samples between groups. **(A)** LDA value distribution histogram. The figure shows the species whose LDA Score is higher than the set value (the default is 3.0). The length of the histogram represents the impact of different species, and different colors represent species in different groups. **(B)** Evolutionary branch graph of LEfSe analysis. The circle of radiation from the inside to the outside represents the taxonomic rank from phylum to species. Each small circle at a different classification level represents a classification at that level. The diameter of the small circle is proportional to the relative abundance. The coloring principle is to color the species with no significant difference uniformly as yellow. Other different species are colored according to the group with the highest abundance of the species. Different colors represent different groups, and the nodes with different colors represent the microflora that plays an important role in the group represented by the color.

Line discriminant analysis effect size analysis identified numerous bacteria with significantly different abundances between CON, LRPG, MRPG, and HRPG (Figure 3). Bacteria with LDA scores greater than 3 were speculated to have different abundance across the four dose groups. Here, MRPG cows had a greater abundance of *Lachnospiraceae_UCG-001*, *Lachnospiraceae_UCG-008*, *Escherichia_Shigella*, *Rumini- clostridium_9*, and *Helicobacter*. HRPG cows had a higher abundance of *Ruminococcaceae_UCG-005*, *Sphingomonas*. CON cows had a greater abundance of *Dorea*, *Prevotella_1*, *Prevotellaceae_UCG-003*. Furthermore, we found that the overall abundance of bacteria in the *Firmicutes* phylum was greater in MRPG, while that of *Bacteroidetes* was greater in CON group.

### Fecal metabolomics profiling

**FIGURE 4:**
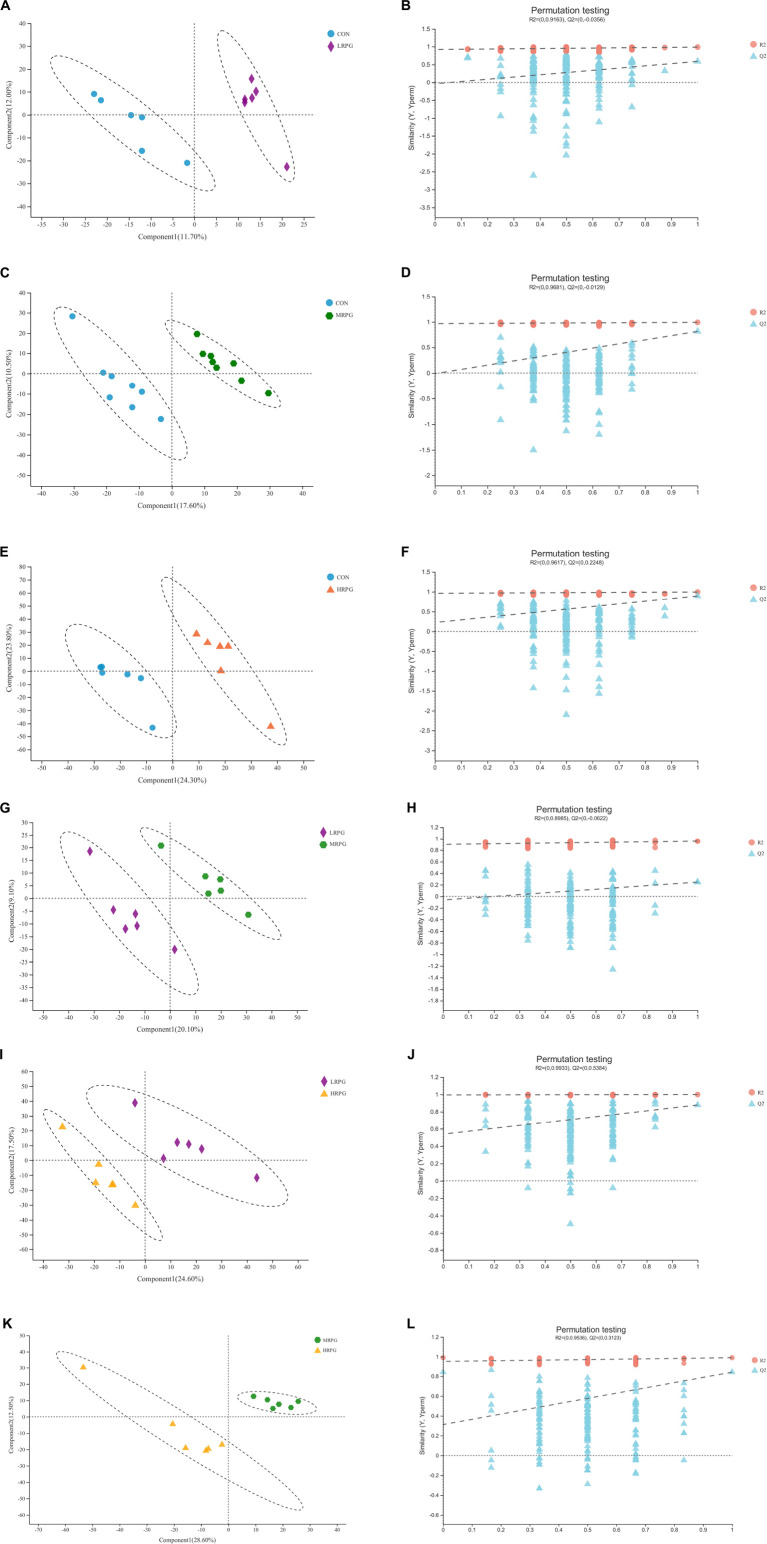
PLS-DA score plots and corresponding validation plots derived from the LC-MS metabolite profiles of rumen samples for cows fed increasing doses of RPG supplementary in their diets. PLS-DA score plots and corresponding validation plots (respectively) for: **(A,B)** the CON group vs. LRPG group, **(C,D)** the CON group vs. MRPG group, **(E,F)** the CON group vs. HRPG group, **(G,H)** the LRPG group vs. MRPG group, **(I,J)** the LRPG group vs. HRPG group, and **(K,L)** the MRPG group vs. HRPG group. CON, control group, a basal diet; LRPG, low RPG, a basal diet plus 200 g/d RPG; MRPG, medium RPG, a basal diet plus 350 g/d RPG; HRPG, high RPG, a basal diet plus 500 g/d RPG.

The PLS-DA score plot of fecal samples revealed significant separation between comparison groups, and the permutation testing R^2^ between pairwise comparisons was all greater than 0.89 (Figure 4), which indicates that the model had satisfactory validity and could be used to identify the difference between the two groups.

**FIGURE 5:**
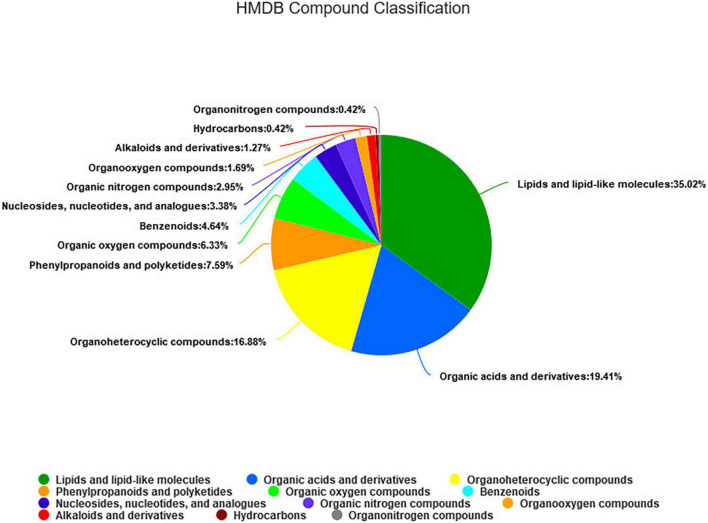
The HMDB compound classification of 295 identified metabolites. According to the number of metabolites, the name of selected HMDB hierarchy (superclass) and the percentage of metabolites are displayed from high to low. Different colors in each pie chart represent different HMDB classification, and the area represents the relative proportion of metabolites in the classification.

As shown in Figure 5, a total of 295 metabolites (*P* < 0.05) were identified and quantified in the fecal samples (Supplementary Table 3) according to querying the Human Metabolome Database (HMDB).^2^ Among them, 35.02, 19.41, and 16.88% of the differential metabolites were classified into lipids and lipid-like molecules, organic acids and derivatives, and organ heterocyclic compounds, respectively. Through further screening, a total of 45 differential metabolites (*P* < 0.05, VIP > 2) were obtained from the pairwise comparisons between the four groups. Among them, 13, 22, 17, 9, 6, and 11 metabolites (*P* < 0.05, VIP > 2) were significantly different between CON vs. LRPG, CON vs. MRPG, CON vs. HRPG, LRPG vs. MRPG, LRPG vs. HRPG, and MRPG vs. HRPG, respectively. Meanwhile, 11, 9, 5, 4, 3, 1, and 1 metabolites were classified into lipids and lipid-like molecules, organ heterocyclic compounds, organic acids and derivatives, phenylpropanoids and polyketides, organic oxygen compounds, nucleosides, nucleotides, and analogs, organooxygen compounds, respectively (Supplementary Table 4).

**FIGURE 6:**
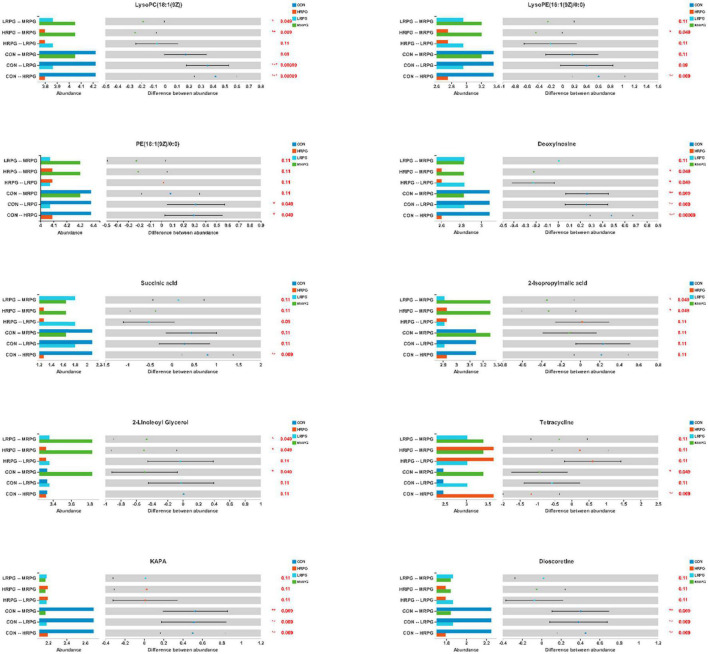
Differential bar graph of differential metabolites. The *X*-axis of the left histogram represents the average relative abundance of a certain metabolite in different groups, the *Y*-axis represents the group categories of pairwise comparison, and different colors represent different groups. The middle region is within the confidence interval set, and the value corresponding to the dot represents the difference of the average relative abundance of metabolites in the two groups. The color of the dot is the group color with a large proportion of metabolites. The interval of type I on the dot is the upper and lower limit of the difference value. The right-most value is *P*, *0.01 < *P* 0.05, **0.001 < *P* 0.01, ***P < 0.001.

Here, we presented histograms of 11 major differential metabolites between each pair of comparison groups (Figure 6). CON cows had the greatest concentrations of KAPA, succinic acid, and some metabolites of lipids, like LysoPC(18:1(9Z)), LysoPE(16:1(9Z)/0:0), dioscoretine, and PE(18:1(9Z)/0:0). In contrast, MRPG cows had the highest concentrations of 2-isopropylmalic acid (2-iPMA) and 2-linoleoyl glycerol (2-LG). In addition, MRPG showed the highest concentrations of multiple metabolites in the paired comparison between the three RPG supplementation groups, like LysoPC(18:1(9Z)), LysoPE(16:1(9Z)/0:0), and PE(18:1(9Z)/0:0). While HRPG cows had a greater concentration of tetracycline than CON and the lowest concentrations of thymine and deoxyinosine.

### Correlations between the fecal microbiome and metabolome

**FIGURE 7:**
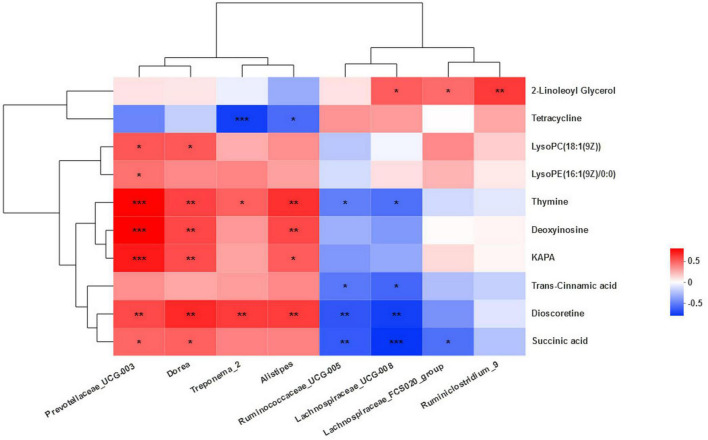
Correlation between the microbiota and metabolites. Cells are colored based on Spearman’s correlation coefficient: positive correlations are shown in red and negative correlations in blue. ***Means strongly significant relationship (*P* < 0.001), **means extremely significant relationship (0.001 < *P* < 0.01), and *means significant relationship (0.01 < *P* < 0.05).

A correlation analysis between the differential bacteria and major differential metabolites was conducted to investigate the potential co-occurrences as displayed with a correlation heat map. *Prevotallaceae_UCG-003* was strong positively associated (*r* > 0.67, *P* < 0.001) with thymine, deoxyinosine, and KAPA and positively associated (*r* > 0.43, *P* < 0.04) with succinic acid, dioscoretine, LysoPE(16:1(9Z)/0:0), and LysoPC(18:1(9Z)). *Dorea* was positively associated (*r* > 0.48, *P* < 0.02) with KAPA, deoxyinosine, thymine, succinic acid, dioscoretine, LysoPC(18:1(9Z)). *Treponema_2* was positively associated (*r* > 0.47, *P* < 0.02) with dioscoretine and thymine, but strong negatively associated (*r* = −0.64, *P* = 0.001) with tetracycline. *Alistipes* was positively associated (*r* > 0.49, *P* < 0.02) with dioscoretine, thymine, deoxyinosine, and KAPA and negatively associated (*r* = −0.48, *P* = 0.02) with tetracycline. *Ruminococcaceae_UCG-005* was negatively associated (*r* < −0.42, *P* < 0.04) with dioscoretine, succinic acid, and thymine. *Lachnospiraceae_UCG-008* was also negatively associated (*r* < −0.47, *P* < 0.02) with dioscoretine, succinic acid, and thymine, but positively associated (*r* = 0.48, *P* = 0.02) with 2-LG. *Lachnospiraceae_FCS020_group* was negatively associated (*r* = −0.47, *P* = 0.02) with succinic acid and positively associated (*r* = 0.45, *P* = 0.03) with 2-LG. *Ruminiclostridium_9* was also positively correlated (*r* = 0.58, *P* = 0.004) with 2-LG (Figure 7).

## Discussion

It is increasingly clear that the composition of mammalian gut microbial communities is essentially driven by diet. These microbiota form intricate symbiotic relationship with their hosts, which has profound implications for overall health (La Reau et al., 2016). The variable presence of microorganisms exploiting diet-dependent metabolic pathways may be key to understanding the variable host response to specific dietary components and susceptibility to disease (Zeevi et al., 2015). Perturbations of gut microbiota composition or functions may play a crucial part in the development of health associated with altered metabolism (Velagapudi et al., 2010). The gut microbiota has recently been identified as an environmental factor that may promote metabolic diseases. The gut microbiota not only influences host energy and lipid metabolism but also highlights its role in the development of metabolic diseases (Velagapudi et al., 2010). Nevertheless, NEB is widely regarded as the pathological basis of metabolic disorders such as ketosis and fatty liver disease. Recent findings revealed that RPG significantly alleviated NEB in cows, significantly reduced NEB-related serum biochemical indexes such as NEFA and BHBA, and changed lipid metabolism in the body. However, to the best of our knowledge, no current data establishes associations between fecal microbiota shifts and doses in RPG. The theory about which (and how) bacteria affect gut metabolism after RPG supplementation is not fully clear yet (Lourenco et al., 2020). Although Zhang et al. (2019) have provided an overview of the ileal microflora under RPG supplementation, the effects of RPG on gut microbiota and metabolism need to be further studied. A more complete characterization of dairy cows’ fecal bacterial community composition afforded by combined analysis of metabolome and 16S can also help address current research gaps concerning the effects of RPG on the changes of microflora and its metabolism in the hindgut of dairy cows. Inspired by Xue et al.’s 2020 research which assessed potential regulatory mechanisms of milk protein production at both the rumen microbiome and host levels by analyzing rumen metagenomics and metabolomics as well as serum metabolomics, we studied microbial changes in the hindgut at different doses of RPG supplementation by combining fecal 16S rRNA gene amplicon sequencing with metabolomics analysis. Because a massive outflow of fermentable materials from the rumen to the hindgut is considered to lead to post-rumen fermentation in the hindgut, we believe that the fecal microbiota not only partly reflects ruminal microbiota but also represents hindgut microbiota (Uchiyama et al., 2020). Furthermore, the method of collecting rumen contents is invasive and may cause damage to the health of dairy cows. Thus, considering animal welfare, the fecal microbiota is part of the final product of digestion, which could be used as an effective and non-invasive balance diagnosis of gastrointestinal tract health (Hagey et al., 2019). In this study, we used the Illumina-MiSeq sequencing method to study the changes in the microbial composition and metabolism of Holstein cow feces after supplementing RPG.

### Changes of gut microbes at the phylum level after rumen-protected glucose supplementation

*Firmicutes* and *Bacteroidetes* in particular play a critical role in the microbial ecology of the mammalian gut, including the bovine gut (Shanks et al., 2011). Clustering analysis showed that the *Firmicutes* and *Bacteroidetes* were the most dominant phyla in the total sequences, which was consistent with studies by Xie et al. (2020). Importantly, in this study, the composition and structure of the fecal microbial community were affected by RPG. At the phylum level, the relative abundance of *Firmicutes* in the feces of dairy cows supplemented with RPG increased significantly, while the *Bacteroidetes* decreased significantly. The intestinal microflora is usually controlled by bacteria in Firmicutes and Bacteroidetes (La Reau et al., 2016). The changes in relative abundance of *Bacteroidetes* and the *Firmicutes* bacterial divisions are associated with obesity. It is shown that obesity is positively correlated with Firmicutes (Turnbaugh et al., 2006). The change of phylum level in this study indicates that RPG supplement may relieve lipolysis through the regulation of core microflora, so as to exert an effect on fat deposition in the body. Other bacterial phyla were not significantly different among the groups, a plausible reason for this was that the shared microflora was involved in housekeeping functions, thus masking the role of RPG.

### Changes of gut microbes at the genus level after rumen-protected glucose supplementation

*Ruminococcaceae_UCG-005*, *Rikenellaceae_RC9_gut_group*, *Ruminococcaceae_UCG-010*, and *Bacteroides* were the core bacteria, in line with the report by Li et al. (2020), which also revealed that the dominant genera were *Ruminococcaceae_UCG-005* and *Rikenellaceae_RC9_gut_group* as well as *Bacteroides* observed in feces of dairy cows in the spring season, our study was conducted in the fall, similar to spring. After different doses of RPG supplementation, there were significant differences in the microbiota of feces between groups. The fecal abundances of *Ruminococcae_UCG-005*, *Ruminiclostridium_9*, *Lachnospiraceae_FCS020_group*, and *Lachnospiraceae_UCG-008* in MRPG group were significantly higher than that of CON or LRPG. They belong, respectively, to the *Ruminococcaceae* and *Lachnospiraceae* families. The *Lachnospiraceae* and *Ruminococcaceae* are two of the most abundant families from the order *Clostridiales* found in the mammalian gut environment (Biddle et al., 2013; Mancabelli et al., 2017); both families include cellulose- and hemicellulose-degrading bacteria and members contributing to butyrate production (Lourenco et al., 2020). Butyrate is absorbed by the gastrointestinal tract epithelium. Here, it is converted to β-hydroxybutyrate and acetoacetate and used as energy substrates for the epithelial cells. That could be beneficial to the energy metabolism and development of the gastrointestinal tract in cows (Kamke et al., 2016), that may protect healthy cows from chronic intestinal inflammation (Lepage et al., 2011) and have been associated with the maintenance of gut health and a healthy state of dairy cows (Biddle et al., 2013; Mancabelli et al., 2017). More specifically, *Ruminiclostridium_9* belongs to the *Ruminococcaceae* family and is associated with the release of inflammatory and cytotoxic factors from the gut for maintenance of a stable intestinal microecology (Xiao et al., 2019). The *Lachnospiraceae* family is known to contribute to volatile fatty acid production, especially butyrate, from diverse polysaccharides (Seshadri et al., 2018). Reduction in the abundance of butyrate-producing *Lachnospiraceae*, which is beneficial for the intestinal barrier, was involved in the formation of visceral hypersensitivity (Zhang et al., 2018). Furthermore, those short-chain fatty acids are known for their anti-inflammatory properties and may also prevent against the development of metabolic diseases (Nguyen et al., 2019). Together, the addition of RPG, especially MRPG, could regulate the relative abundances of intestinal health-related bacteria, and play a positive role in reducing intestinal inflammation and preventing related metabolic diseases.

Other abundant differential genera including *Prevotellaceae_UCG-003*, *Alistipes*, and *Dorea* showed the highest levels in CON group. Previous findings revealed that different *Prevotella spp.* can selectively utilize carbohydrates and proteins from diet to produce succinate and acetate (Stevenson and Weimer, 2007). However, although *Prevotella* have this capability of breaking down different carbohydrates, one of their most significant roles is in the protein and peptide breakdown (Lourenco et al., 2020). This genus plays an important role in normal intestinal metabolism and is also an important substance to maintain intestinal health (Xue et al., 2020). Similar evidence was observed in previous investigations, which highlighted that anaerobic organism such as the abnormal increase of *Prevotellaceae* abundance exacerbated the occurrence of inflammation (Elinav et al., 2011). *Alistipes* is a relatively new genus of bacteria, which was also classified as a member of the phylum *Bacteroidetes* (Kim et al., 2011). Although at a low rate compared to other genus members of the *Bacteroidetes* phylum, which are highly relevant in dysbiosis and disease (Parker et al., 2020). In a previous study, the percentage of genera *Alistipes* was significantly decreased in the fecal microbiota of colorectal cancer patients by 454 pyrosequencing (Li et al., 2020). In contrast, other studies indicate *Alistipes* is pathogenic in colorectal cancer and is associated with mental signs of depression (Parker et al., 2020). Meanwhile, dietary change affected the abundance of *Alistipes*, which is a bile-tolerant bacterium (David et al., 2014). Notably, in our study, there was a significant negative correlation between *Alistipes* and tetracycline concentration involved in bile synthesis, that may be related to the location of the farms and the feeding operation. The fecal microbiota of calves is rich, diverse, and potentially highly variable between farms (Weese and Jelinski, 2017). The feeding operation is a more important determinant of the cattle microbiome than in the geographic location of the feedlot (Shanks et al., 2011). *Dorea*, an intestinal bacterium that produces gas with carbohydrates, experienced a marked increase in irritable bowel syndrome. In the feces of patients with non-alcoholic fatty liver disease, the abundance of *Dorea* increased while that of *Ruminococcaceae* decreased (Raman et al., 2013). Similar to the change of bacteria in CON in this study, this indicated that cows in the control group may have risks such as impaired intestinal immunity and lipid metabolism disorder. The three bacteria mentioned above had the highest levels in the control group, the result corresponded with the downregulation of amino acid metabolism and associated intestinal inflammation in dairy cows.

### Connection between microbe and metabolites

The change in the microbial community was probably caused by an altered metabolism of the host linked to a modified secretion of host metabolites into the gut lumen (Tilocca et al., 2017). A previous study suggested that the fecal bacterial community composition correlated significantly with fecal starch concentrations, largely reflected in changes in the *Bacteroidetes*, Proteobacteria, and *Firmicutes* populations (Shanks et al., 2011). As a result, a mechanism through which diet can influence immune responses is the gut microbiome, which is emerging as a critical contributor in numerous human diseases (Cignarella et al., 2018). The interactions between intestinal bacteria and the host immune system are mediated either via direct contact between bacteria and the innate immune system (e.g., toll-like receptors, NOD2 receptors) or through microbial metabolites. These metabolites can be produced directly by bacteria (e.g., vitamins, SCFA) or are primary host metabolites that are converted through bacterial enzymes into secondary metabolites (e.g., conversion of primary to secondary bile acids) (Suchodolski, 2016). The above contents suggest a higher concern of the response of fecal metabolites to RPG supplementation. Ultra-performance liquid chromatography (UPLC) coupled with high-resolution mass spectrometry (MS) allows reproducible measurements of a wide range of metabolites in a complex sample (Marcobal et al., 2013).

### Dominant gut microbial metabolites of control group, a basal diet without rumen-protected glucose, after rumen-protected glucose supplementation

LysoPC(18:1(9Z)), PE(18:1(9Z)/0:0), LysoPE(16:1(9Z)/0:0), and dioscoretine belong to lipids and lipid-like molecules. Compared with the three RPG groups, the three metabolites were at the highest levels in CON group. LysoPC(18:1(9Z)) is a kind of lysophosphatidylcholines (LysoPC) that belong to glycerophospholipids, resulting from the partial hydrolysis of phosphatidylcholines, involved in choline metabolism in cancer and the glycerophospholipid metabolism pathway. LysoPC is a strong proinflammatory mediator, and it has been suggested to be mediated via the platelet-activating factor (PAF) receptor (Oestvang et al., 2011). LysoPC initiate and contribute to inflammation through the activation of phospholipase a2 enzymes and the consequent release of amino acid, which is a precursor for the proinflammatory hormones, the eicosanoids (Oestvang et al., 2011). LysoPCs are the major components of ox-low density lipoprotein which play dual functions in the cardiovascular disease (Nuli et al., 2019). LysoPC can activate endothelial cells during early atherosclerosis and can stimulate phagocyte recruitment when they were released by apoptotic cells (Li et al., 2018). Another glycerophosphate PE(18:1(9Z)/0:0) that positively regulate autophagy and longevity (Rockenfeller et al., 2015) is one kind of glycerophosphoethanolamines. Phosphatidylethanolamine (PE) is a class of phospholipids found in biological membranes; it can be found in all living organisms. PE is the second-most abundant phospholipid in mammalian membranes ranging from 20 to 50% (Nuli et al., 2019). LysoPE(16:1(9Z)/0:0) is a kind of lysophosphatidylethanolamine (LysoPE) that belongs to glycerophospholipids, resulting from the partial hydrolysis of PE. Metabolites such as LysoPE were related to the amount of fatty acid in feces (Lee et al., 2013). As stated before, Nuli explored the mechanisms underlying the potential characteristic metabolite changes during different glycemic stages and demonstrated that significantly changed metabolites LysoPC and PE between the normal group and the impaired glucose regulation patients were involved in glycerophospholipid metabolism (Nuli et al., 2019). Together, the results showed that the changes of glycerophosphate in the intestine could reflect the state of glucose metabolism in the body. RPG can down-regulate lipid metabolism and indirectly regulate the body’s glucose metabolism.

### Dominant gut microbial metabolites of rumen-protected glucose groups after rumen-protected glucose supplementation

2-IPMA is mainly involved in carbohydrate metabolism and amino acid metabolism. 2-iPMA, an intermediate compound in the leucine biosynthesis pathway, is synthesized from α-ketoisovalerate and acetyl-coenzyme A, a reaction catalyzed by 2-iPMA synthase (Suzuki et al., 2007). Moreover, 2-iPMA possesses superior Al(III)-ion detoxification ability. 2-iPMA secreted from the yeast cells chelates Al ions and prevents them from entering the cells, resulting in Al tolerance (Suzuki et al., 2007). MRPG regulated amino acid metabolism and carbohydrate metabolism and may help alleviate heavy metal poisoning. MRPG interacted amino acid metabolism and carbohydrate metabolism and may help alleviate heavy metal poisoning. Similarly, MRPG significantly increases the concentration of 2-LG. In mammals, biosynthesis, degradation, and metabolism of these bio-active lipids like 2-arachidonoylglycerol (2-AG) and 2-LG intertwine and form a complicated biochemical pathway to affect the mammal neuromodulation of the central nervous system and also other physiological processes in most peripheral organs and non-nervous tissue cells (Yuan et al., 2016). For instance, 2-LG significantly inhibited the inactivation of 2-AG in neuronal and basophilic cells (Murataeva et al., 2016). After MRPG supplementation, some intestinal metabolites participated in neuromodulation. HRPG significantly down-regulated abnormal nucleotide metabolism. Deoxyinosine is an abnormal nucleoside and has hypoxanthine as its base moiety (Murataeva et al., 2016). Deoxyinosine occurs in DNA either by oxidative deamination of a previously incorporated deoxyadenosine residue or by misincorporation of deoxyinosine triphosphate from the nucleotide pool during replication (Yoneshima et al., 2016). Deoxyinosine triphosphate induces cell growth arrest and DNA instability in mammalian cells. The individual nucleobases in feces were analyzed by HPLC after hydrolysis using HClO, all the individual nucleobases were highly digestible. Uracil showed the highest digestibility (on average 96.8%), whereas thymine showed the lowest digestibility (Mydland et al., 2008). Considering that, it was not surprising that HRPG may also regulate related bacteria to improve the digestibility of thymine, for example, significantly negatively correlated *Ruminococcaceae_UCG-005* and *Lachonospiraceae_UCG-008*. However, additional targeted investigations are necessary to explore the mechanism. Collectively, MRPG improved the carbohydrate metabolism and amino acid metabolism pathway in dairy cows and changed the neuromodulation-related metabolites. HRPG supplementation down-regulated abnormal nucleotide metabolism and reduced the concentration of harmful metabolites in nucleotide metabolism pathway.

### Correlations between microbial communities and fecal metabolites

The results of the correlation analysis showed that *Prevotellaceae_UCG-003* and *Dorea* were positively correlated with most of the major differential metabolites. The interactions between microbial taxa and functions with microbial metabolites suggest that the *Prevotellaceae* family may be crucial contributors to microbial metabolites including amino acids and carbohydrates. This observation was consistent with the report by Xue et al. (2020). Gut microbiota is the predominant source of luminal succinate (Faith et al., 2014). Succinate is a gut microbiota-derived metabolite with a key role in governing intestinal homeostasis and energy metabolism (Fernández-Veledo and Vendrell, 2019). The *Prevotella* species, along with *Succinimonas amilolytica*, act as a succinate-producing bacterium in the bovine rumen (Bryant et al., 1958). Therefore, we retain that the positive correlation could be explained. Even though, the available evidence suggested a link between dysbiosis, succinate accumulation in gut, and inflammation (Fernández-Veledo and Vendrell, 2019). In other words, succinic acid in the intestine was advantageous in certain concentrations. The human (mammalian) biotin cycle in humans is complex. Humans do not synthesize biotin but must obtain it from the diet or, perhaps, from gut bacteria (Beckett, 2018). 7-Keto-8-aminopelargonic acid synthase (KAPA synthase) is the enzyme that catalyzes the first committed step of the biotin synthesis pathway (Fan et al., 2015). Biotin is an essential cofactor for enzymes that function in the carboxylation, decarboxylation, and trans-carboxylation reactions found in processes such as fatty acid biosynthesis, gluconeogenesis, and amino acid metabolism (Fan et al., 2015; Beckett, 2018). *Prevotellaceae_UCG-003*, *Dorea*, and *Alistipes*, which were positively related, may regulate glucose metabolism and amino acid metabolism through the important role of this enzyme. Overall, *Prevotellaceae* and *Dorea* were important contributors of differential metabolites. Succinic acid and some lipids were important differential metabolites in this study. *Bacteroidetes* were positively correlated with major differential metabolites, while *Firmicutes* were negatively correlated with them.

## Conclusion

This study provides the first in-depth understanding of changes in hindgut microbiota, its metabolites and their relationships by combining high-throughput microbiomics and untargeted metabolomics after RPG supplementation in early lactation dairy cows. RPG supplement may relieve lipolysis through the regulation of core microflora, so as to exert an effect on fat deposition in the body, especially MRPG, could regulate the relative abundances of intestinal health-related bacteria, and play a positive role in reducing intestinal inflammation and preventing related metabolic diseases. MRPG improved the carbohydrate metabolism and amino acid metabolism pathway in dairy cows and changed the neuromodulation-related metabolites. HRPG supplementation down-regulated abnormal nucleotide metabolism and reduced the concentration of harmful metabolites in nucleotide metabolism pathway. Overall, our study contributes to an increased understanding of how the RPG supplementation affects the fecal microbiota functions. Our work also provides insights into the influence of RPG on gut metabolism. Further research is needed to understand how RPG influences fat metabolism and glucose metabolism by lipidomics and glycomics, so as to deeply reveal the metabolic mechanism of RPG alleviating NEB.

## Data availability statement

The datasets presented in this study can be found in online repositories. The names of the repository/repositories and accession number(s) can be found in the article/Supplementary material.

## Ethics statement

The animal study was reviewed and approved by the Animal Ethics Committee of the Chinese Academy of Agricultural Sciences (Beijing, China) (Approval Number: IAS2019-54).

## Author contributions

YPW conceived, designed, and performed the experiment and drafted the manuscript. XN and YZ participated in the editing of the manuscript. YUW and MC were involved in the animal experiment and sample analysis. QL, BX, and LJ supervised and provided continuous guidance for the experiment. All authors contributed to the article and approved the final manuscript.

## Footnotes

1
http://www.ncbi.nlm.nih.gov/Traces/sra/

2
http://www.hmdb.ca

## Supplementary material

Supplementary Figure 1 Sample Shannon index curve.

Supplementary Figure 2 Sample rank-abundance curve.

Supplementary Table 1 Nutrition composition of the diet fed to early lactation dairy cows.

Supplementary Table 2 Ultra-performance liquid chromatography (UPLC) elution gradient.

Supplementary Table 3 Main microbiota (accounting for ≥ 0.05% of the total sequences in at least one of the samples) in the four groups (abundance of the phylum is expressed as a percentage).

Supplementary Table 4 List of differential metabolites.
